# Application of Surfactant Micelle-Entrapped Eugenol for Prevention of Growth of the Shiga Toxin-Producing *Escherichia coli* in Ground Beef

**DOI:** 10.3390/foods6080069

**Published:** 2017-08-16

**Authors:** Tamra N. Tolen, Songsirin Ruengvisesh, Thomas M. Taylor

**Affiliations:** 1 Department of Animal Science, Texas A & M University, College Station, TX 77843-2471, USA; ttolen84@tamu.edu; 2 Department of Nutrition and Food Science, Texas A & M University, College Station, TX 77843-2253, USA; songsirin@gmail.com

**Keywords:** plant-derived antimicrobial, micelles, non-O157 STEC, *E. coli* O157:H7, ground beef, beef safety, eugenol

## Abstract

Beef safety may be compromised by O157 and non-O157 Shiga toxin-producing *Escherichia coli* (STEC) contamination. The capacity of surfactant micelles loaded with the plant-derived antimicrobial eugenol to reduce STEC on beef trimmings that were later ground and refrigerated for five days at 5 ± 1 °C was tested to determine their utility for beef safety protection. STEC-inoculated trimmings were treated with free eugenol, micelle-encapsulated eugenol, 2% lactic acid (55 °C), sterile distilled water (25 °C), or left untreated (control). Following treatment, trimmings were coarse-ground and stored aerobically at 5 ± 1 °C. Ground beef was then sampled for STEC immediately post-grinding, and again at three and five days of storage. STEC minimum inhibitory concentrations (MICs) in liquid medium for free eugenol and 1% sodium dodecyl sulfate (SDS)-loaded micelles were 0.5% and 0.125%, respectively. STEC numbers on beef trimmings treated by sterile water (6.5 log_10_ CFU/g), free eugenol (6.5 log_10_ CFU/g), micelle-loaded eugenol (6.4 log_10_ CFU/g), and lactic acid (6.4 log_10_ CFU/g) did not differ compared to untreated controls (6.6 log_10_ CFU/g) (*p* = 0.982). Conversely, STEC were significantly reduced by refrigerated storage (0.2 and 0.3 log_10_ CFU/g at three and five days of storage, respectively) (*p* = 0.014). Antimicrobial treatments did not significantly decontaminate ground beef, indicating their low utility for beef safety protection.

## 1. Introduction

The O157 and non-O157 Shiga toxin-producing *Escherichia coli* (STEC) are known causes of human foodborne disease, inducing both acute and chronic sequelae ranging from mild diarrheal disease to hemolytic uremic syndrome (HUS), renal failure and potentially death [1]. The U.S. Centers for Disease Control and Prevention (CDC) has estimated 265,000 cases of STEC-associated human foodborne disease occur annually in the U.S.; 36% percent of these are reportedly associated with O157 STEC and the balance the result of the non-O157 STEC [2,3]. In addition to *E. coli* O157:H7, six serotypes of the non-O157 STEC have been declared adulterants in fresh non-intact beef products by the U.S. Department of Agriculture-Food Safety and Inspection Service (USDA-FSIS) [4]. Estimates of the annual financial costs of these pathogens in the U.S. with respect to income loss, medical expenses, and other quantifiable costs have ranged from $280 million to $790 million per annum [5,6].

Cattle have been identified as a reservoir for the STEC, potentially facilitating cross-contamination of beef products during handling of meat [7,8,9]. Fresh beef can be cross-contaminated during the fabrication of beef trimmings destined for use in the production of ground beef [10]. Antimicrobial interventions applied for the decontamination of beef trimmings and/or ground beef have exhibited varying levels of efficacy for STEC reduction. Geornaras et al. [11] reported that the application of antimicrobial interventions on trimmings produced from beef chuck rolls, including 0.1% acidified sodium chlorite and 0.02% peracetic acid, produced reductions in STEC varying from 0.4 to 1.8 log_10_ CFU/cm^2^. Others have reported greater reductions in STEC on trimmings and ground beef following application of lactic acid as a beef safety intervention. Harris et al. [12] reported that STEC were reduced by 1.9 to 2.0 log_10_ CFU/g or 2.1 to 2.5 log_10_ CFU/g following application of 2% or 4% lactic acid, respectively. This group recently reported that application of acetic acid (2% or 4%) and acidified sodium chlorite (1000 ppm) produced reductions in STEC on beef trimmings ranging from 0.3 to 0.5 log_10_ CFU/g (*p* < 0.05) [13]. Nonetheless, researchers described STEC reductions as being of little practical utility for food safety protection.

In addition to exploring the use of traditional antimicrobials for reducing STEC on fresh beef products, researchers have investigated plant-derived antimicrobials (PDAs), including essential oils and their components, for beef safety protection. Application of commercial herb extract-containing products to STEC-inoculated beef trimmings, two rounds of grinding, and up to seven days’ refrigerated (4 °C) storage, produced reductions of 0.1 to 0.2 log_10_ CFU/g in STEC numbers [14]. More recently, researchers reported that the application of 0.05% or 0.1% rutin, or 0.1% or 0.2% resveratrol, did not reduce *E. coli* O157:H7 in ground beef patties during refrigerated storage, but did enhance reductions of the pathogen upon subsequent cooking to an internal temperature of 65 °C [15]. The use of encapsulation strategies, such as surfactant micelles, has been described as providing opportunity for enhanced transport and delivery of entrapped compounds to foodborne microbes in both animal- and plant-based foods [16,17,18]. The use of micelle-encapsulated eugenol for reducing O157 or non-O157 STEC on fresh beef has not, to the authors’ knowledge, been reported in the scientific literature.

Application of such antimicrobial interventions may be useful for beef safety protection in the manufacture of natural beef products. Hence, the objectives of this study were to: (i) quantify the capability of free and micelle-encapsulated eugenol to produce statistically significant reductions in numbers of inoculated STEC on beef trimmings prior to beef grinding when applied at concentrations not likely to produce long-term negative impacts on consumer acceptability; and, (ii) determine the capacity of antimicrobial treatments applied to beef trimmings to inhibit STEC growth and/or reduce STEC numbers on ground beef during refrigerated storage post-treatment.

## 2. Materials and Methods

### 2.1. Microorganisms and Revival Procedures

STEC isolates belonging to serotypes O157:H7, O26:H11, and O121:H19, resistant to 100 µg/mL rifampicin (Rif^R^), were provided by J.B. Luchansky (USDA Agricultural Research Service, Wyndmoor, PA, USA) and preserved at −80 °C upon receipt in the culture collection of the Food Microbiology Laboratory (Department of Animal Science, Texas A & M University, College Station, TX, USA). Isolates were revived by duplicate identical passages in sterile tryptic soy broth (TSB; Becton, Dickinson and Co., Franklin Lakes, NJ, USA), followed by incubation at 35 ± 1 °C for 24 h. Following revival, isolates were individually streaked onto Petri dishes containing MacConkey Agar (Becton, Dickinson and Company, Sparks, MD, USA) supplemented with 100 µg/mL rifampicin (Sigma-Aldrich Co., St. Louis, MO, USA), and incubated for 24 h at 35 ± 1 °C to verify Rif^R^ capacity, as well as organisms’ ability to utilize lactose and decompose bile salts. Well-isolated colonies were picked with sterile needles and slant cultures of each isolate were prepared on tryptic soy agar (Becton, Dickinson and Company) for later use during experimental trials. Slants were layered with sterile mineral oil and stored at 5 ± 1 °C until being prepared for use via the revival procedure described above.

### 2.2. Preparation of Surfactant for Maximum Non-Inhibitory Concentration (MNIC) Determination

Sodium dodecyl sulfate (SDS; CAS #151-21-3) was purchased from Sigma-Aldrich Co.; a 30% (*w/v*) stock was prepared by mixing 12.0 g SDS in 40.0 mL sterile distilled water in a sterile container. SDS has been previously reported to exert antimicrobial activity against members of the STEC at concentrations as low as 0.5% SDS [19]. Thus, to determine whether SDS exerted antimicrobial effects against STEC at experimental concentrations on its own, each STEC isolate was individually inoculated into a 96-well microplate and treated with 2-fold serially diluted SDS. STEC isolates were individually serially diluted prior to loading into sample microplate wells to obtain a target 5.0 log_10_ CFU/mL inoculum in double-strength TSB (2× TSB; 60 g TSB powder/liter water); 100.0 µL of each isolate was added into surfactant solution-containing wells. Surfactant was added first (200.0 µL), and then 100.0 µL was extracted and loaded into the adjacent well containing 100.0 µL sterile distilled water, according to the method of Pendleton et al. [20]. This process was completed as needed to produce sufficient wells containing 2-fold serially diluted SDS before wells were loaded with a STEC isolate. Loaded, inoculated plates were sealed with plate sealers (Thermo-Fisher Scientific, Waltham, MA, USA) and optical density at 630 nm (OD630) read on a UV/Visible Spectrophotometer (BioTek^®^ Instruments, Inc., Winooski, VT, USA). In addition to the experimental wells described, microplate wells containing identically prepared and loaded solutions of SDS, 2× TSB, and sterile water, but no STEC, were prepared and read at OD630. This was completed in order to provide for baseline adjustment in experimental data to allow for identification of minimum inhibitory concentrations (MICs) of SDS and eugenol, using the method of Brandt et al. [21]. STEC-inoculated wells bearing >0.05 change in OD630 following incubation were labeled as non-inhibitory with respect to SDS concentration applied. Wells with the highest content of SDS not inhibitory to STEC, the maximum non-inhibitory concentration (MNIC), were chosen to prepare micelles for subsequent MIC experiments for STEC.

### 2.3. Preparation of Eugenol-Loaded Micelles and Unencapsulated Eugenol for MIC Determination

Working solutions from a 70% eugenol stock prepared in 95% ethyl alcohol (EtOH) were diluted in 95% EtOH to desired eugenol concentrations; eugenol and EtOH were purchased from Sigma-Aldrich Co. The working stock was stored at 5 ± 1 °C in a foil-wrapped container until ready for use. Following calculation of required eugenol and surfactant for micelle generation [16], eugenol in alcohol and surfactant in sterile water were mixed and brought to volume with sterile distilled water as required. Mixtures were stirred at room temperature to form micelles. Based on results from experiments detailed above (Section 2.2), 20 mL micelle volumes were prepared to contain varying concentrations of eugenol. Double-strength micelle solutions (max. concentrations: 2.0% SDS ± 2.0% eugenol) were prepared and filtered through a 0.45 µm cellulose acetate filter. Non-encapsulated eugenol was prepared in EtOH and sterile distilled water to identical concentrations as those prepared for eugenol-loaded SDS micelles.

Determination of STEC isolate-specific MICs for free and micelle-loaded eugenol was completed according to published methods [16]. Microplate wells containing individual STEC isolates were exposed to free or encapsulated eugenol at 2-fold serial dilutions and then incubated for 24 h at 35 ± 1 °C. OD630 readings were taken at 0 and 24 h, and baseline corrections made as previously detailed using identically prepared microplate wells loaded with eugenol, 2× TSB, and sterile distilled water, but not inoculated with STEC. Wells bearing <0.05 change in OD630 (<0.05 ∆OD630) after baseline correction were identified as inhibitory for the inoculated STEC isolate. The lowest concentration of free or encapsulated eugenol-producing inhibition was identified as the MIC for each STEC isolate. To verify the maximal load of EtOH contacting STEC in wells was not inhibitory to STEC on its own, a separate set of wells containing only EtOH at maximal calculated concentration was tested alongside other experimental wells. Finally, positive and negative controls were incorporated, bearing STEC plus growth medium only (positive control) or growth medium and sterile water but no STEC inoculum (negative control).

### 2.4. Application of Eugenol-Loaded Micelles to STEC-Inoculated Beef Trimmings and STEC Reductions on Ground Beef Prepared from Treated Trimmings

Frozen beef trimmings (90% lean) were obtained from the Rosenthal Meat Science and Technology Center at Texas A & M University (College Station, TX, USA) and transported to the Food Microbiology Laboratory. Trimmings were thawed at 4 °C for 24 h prior to inoculation and use. On the day of experiment initiation, 1000 g of thawed beef trimmings were inoculated with a three-strain mixture of STEC and sealed in a sterile plastic bag. The STEC mixture was produced by blending equivalent volumes of overnight cultures of revived STEC isolates (Section 2.1) according to previously published methods [22]. Inoculated trimmings were hand-massaged for 1 min in order to distribute inoculum over trimming surfaces, after which beef was left undisturbed in a biological safety cabinet at ambient conditions for 30 min to allow inoculum attachment. Following STEC attachment, a 10 g aliquot was aseptically collected to determine inoculation efficiency by preparation of decimal dilutions in 0.1% peptone diluent and plating onto tryptic soy agar supplemented with 100 µg/mL rifampicin (TSAR)-containing Petri dishes, followed by 24 h incubation at 35 ± 1 °C. Remaining meat was separated into 200 g batches and treated by application of three sprays from a handheld spray bottle (2.4 mL total applied volume) held approximately 30.5–35.6 cm from the trimmings with solutions of sterile distilled water at 25 °C (Water), twice the MIC of free eugenol (2× FreeEug) or three times the MIC of eugenol-loaded SDS micelles (Micelles). These concentrations of free and micelle-loaded eugenol were selected during preliminary trials in which antimicrobial treatments were applied to non-inoculated beef trimmings that were then coarse-ground and stored for 5 days at 5 °C, after which samples were removed and evaluated for residual eugenol (clove essence) odors. These treatments (2× FreeEug, Micelles (3× micelle MIC)) were highest not producing strongly displeasing off-odor of beef samples (data not shown). In addition, beef trimmings were treated by 2% lactic acid pre-warmed to 55 °C (2% LA), a 25 °C sterile distilled water spray (Water), or were left untreated (Control). Spray application, versus immersion application, was selected due to the low likelihood of a small sprayed volume resulting in water uptake in treated beef as compared to immersion application of antimicrobial systems. The USDA forbids addition of water to ground beef beyond a negligible amount (~0.5%) [23].

Following antimicrobial treatment, trimmings were coarse-ground through a 3/8 inch plate using a LEM Products electric meat grinder (Model #781; West Chester, OH, USA). In between the grinding of each batch of treated trimmings, all grinder components were disassembled and residual beef tissue removed by brush-scrubbing while soaking in chlorinated (0.6% FAC) tap water. After all beef was removed, the grinder components and scrubbing brush were soaked for an additional 10 min in chlorinated water (0.6% FAC). Components were then removed and sprayed with 70% EtOH to further sanitize prior to rinsing with water and re-assembly of the grinder. Beef was aseptically separated into 10.0 g samples, randomly assigned to an antimicrobial treatment and storage period, and subjected to assigned antimicrobial intervention treatment. STEC survival was determined immediately after antimicrobial treatment, immediately after grinding, and again at 3 and 5 days of aerobic refrigerated (5 ± 1 °C) storage in order to determine whether STEC reductions would be detected quickly or require additional holding to allow for eugenol release. Samples were placed into stomacher pouches, diluted with 90 mL sterile 0.1% peptone diluent, and stomached for 1 min at 230 rpm. Decimal dilutions were subsequently prepared in 0.1% peptone diluent and surviving STEC enumerated on TSAR following 24 h at 35 ± 1 °C.

### 2.5. Statistical Analysis

MIC experiments were repeated three times in identical fashion (*n* = 3); the lowest inhibitory concentration of free or micelle-loaded eugenol for each STEC isolate across all replicates was identified as the experimental MIC [21]. For beef decontamination experiments, three identical replicates were executed (*n* = 3); log_10_-transformed plate counts were determined from sample plate counts prior to statistical analysis. Reductions in STEC on beef were determined by analysis of variance (ANOVA) and statistically differing means separated by Tukey’s Honestly Significant Differences (HSD) test at α = 0.05.

## 3. Results and Discussion

### 3.1. Minimum Inhibitory Concentration of Eugenol-Loaded SDS Micelles against STEC

The maximum non-inhibitory level of SDS for all STEC isolates tested was 1.0% (Table 1). Additionally, eugenol-loaded micelles were inhibitory to all STEC isolates at 0.125% (*w/v*) eugenol in 1% SDS (Table 1). Micelle-loaded eugenol MICs for STEC are similar to the MICs of 0.15% for eugenol-loaded micelles against *E. coli* O157:H7 strains previously reported by Gaysinsky et al. [24]. The MIC for unencapsulated eugenol against *E. coli* O157:H7, O26:H11, and O121:H19 was 0.5% (*w/v*) (Table 1). Similar MICs for other plant-derived antimicrobials against *E. coli* O157:H7 and other *Enterobacteriaceae* have been reported elsewhere [25,26]. Previous research has also demonstrated enhanced antimicrobial efficacy of plant essential oil components following micelle entrapment in complex food matrices, reportedly resulting from enhanced dispersion of the hydrophobic oils into the aqueous fraction of the food and transport of essential oil components into the membrane of STEC isolates and other bacterial organisms [16,18,25,27,28]. Refrigerated storage is not expected to have significantly inhibited eugenol release by SDS micelles, given previous reports in which a 4.1 log_10_-cycle reduction of *Salmonella Enteritidis* was reported during a 5 min exposure to 1% SDS/levulinic acid micelles at 8 °C [19].

### 3.2. Reduction of STEC on Beef Trimmings by Free and Micelle-Encapsulated Eugenol

The mean number of STEC inoculated onto beef trimmings was 6.6 ± 0.4 log_10_ CFU/g. No treatment produced statistically significant STEC reductions on trimmings versus untreated controls (*p* = 0.902); the greatest numerical reduction in STEC (0.3 log_10_ CFU/g) as compared to the control was observed for 55 °C lactic acid-treated beef trimmings (Table 2). Similar reductions in *E. coli* O157:H7 numbers (0.1 to 0.2 log_10_ CFU/g) have been reported following the treatment of beef trimmings (80% lean) with sprays of either 2% or 4% acetic/lactic acid blends heated to 55 °C [29]. Researchers suggested that inclusion of adipose tissue in trimmings may have compromised antimicrobial activity of applied interventions for pathogen reduction, in addition to impacts of grinding and post-treatment storage. Cutter [14] likewise reported low reductions in *E. coli* O157:H7 following plant extract application in micro-encapsulation systems and subsequent holding.

In contrast to these studies, other studies have reported higher STEC reductions on trimmings when immersed in solutions of traditional antimicrobials. Ellebracht et al. [30] submerged beef trimmings in 95 °C water or 2% lactic acid tempered to 55 °C. Reductions in *E. coli* O157:H7 following hot water and heated acid in this study were 0.5 and 1.1 log_10_ CFU/g, respectively. In a study applying 5% lactic acid via immersion to lean trimmings inoculated with O157 and non-O157 STEC, researchers reported STEC reductions varied from 0.5 to 1.4 log_10_ CFU/cm^2^, depending on whether the acid solution was applied at 25 °C or 55 °C [31]. This group also reported reductions in *E. coli* O157:H7 numbers of 0.5 or 0.7 log_10_ CFU/cm^2^ on inoculated trimmings immersed in 0.1% acidified sodium chlorite or 0.02% peroxyacetic acid, respectively [11]. Finally, research testing 4.4% lactic acid application onto STEC-inoculated beef trimmings compared pathogen reductions when acid was applied by dipping or spraying. STEC reductions obtained by dipping approximated 1.5 log_10_ CFU/g following 20 h post-treatment refrigerated storage, whereas spray-treatment produced only a 0.5 log_10_ CFU/g reduction following post-treatment refrigerated storage [32]. Researchers suggested that dipping in lactic acid solution allowed for enhanced contact of acidulant with STEC on trimmings versus spraying as an explanation for observed differences in STEC reductions [32]. Nevertheless, use of immersion treatment of meat trimmings is of limited utility in the U.S., due in part to sensory impacts and limits on the utility of treatments expected to result in excess water pick-up by meat, thereby resulting in treated products not adhering to the federal standard of identity for such products [23].

### 3.3. Inhibition of STEC on Ground Beef Following Antimicrobial Treatment during Refrigerated Storage

**Figure 1:**
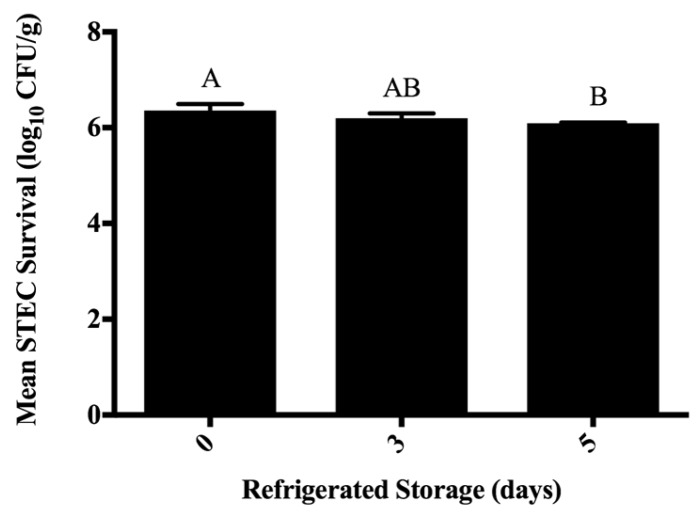
Survival of Shiga toxin-producing *Escherichia coli* (STEC) on ground beef during aerobic refrigerated storage (*p* = 0.014; pooled SE = 0.083). Bars depict means of the main effect of storage period calculated from triplicate identical replicates (*n* = 3); error bars depict one standard deviation from the mean. Columns not sharing letters (A,B) differ at *p* < 0.05. Beef trimmings were ground through a 3/8 inch plate following antimicrobial intervention, separated into 10.0 g samples, and stored for up to five days at 5 ± 1 °C prior to STEC enumeration on Petri dishes containing tryptic soy agar supplemented with 100.0 µg/mL of rifampicin.

**Figure A1:**
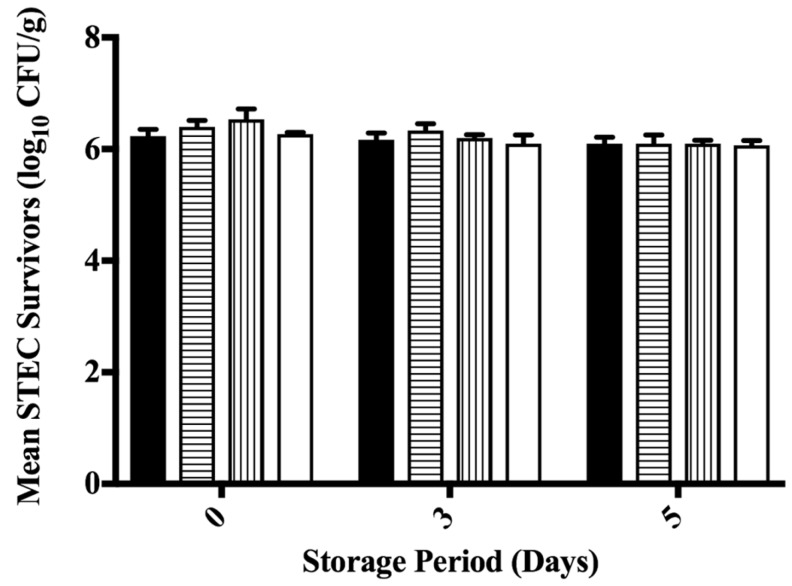
Least squares LS means of Shiga toxin-producing *Escherichia coli* (STEC) as a function of the interaction of antimicrobial treatment x storage period (*p* = 0.812; pooled SE = 0.164). Error bars depict one standard error for treatment mean; means were determined by two-way analysis of variance (ANOVA) and Tukey’s Honestly Significant Differences (HSD) means separation test. Beef trimmings were ground through a 3/8-inch plate following antimicrobial intervention, separated into 10.0 g samples, and stored for up to 5 days at 5 ± 1 °C prior to STEC enumeration on Petri dishes containing tryptic soy agar supplemented with 100.0 µg/mL of rifampicin. Antimicrobial treatments were: 25 °C sterile water spray (filled bars), free eugenol applied at twice the MIC in vitro against STEC (horizontally hashed bars), SDS micelle-loaded eugenol applied at three times in vitro MIC (vertically hashed bars), and 2% lactic acid pre-warmed to 55 °C (open bars).

Analysis of STEC reductions on ground beef at 0, 3, and 5 days of refrigerated storage (5 ± 1 °C) following treatment by water, free or micelle-entrapped eugenol, or 55 °C lactic acid spraying indicated no effect of antimicrobial treatment (*p* = 0.371), and no effect of interaction of antimicrobial treatment with storage duration (*p* = 0.812) (Figure A1 in Appendix A). However, ground beef refrigerated storage period did impact STEC survival on treated ground beef, where STEC counts differed between days 0 and 5 (*p* = 0.014) (Figure 1). Lack of detectable differences in STEC numbers as a function of treatment may have resulted from the homogenization of STEC throughout beef tissue during grinding, or insufficient antimicrobial application despite applying two and three times the respective MICs for free and encapsulated eugenol (Figure 1). Grinding, through the mechanical disruption of beef tissue, exposes previously non-accessible tissue and surfaces to microbes. Likewise, meat grinding would be expected to reduce the efficacy of applied interventions by exposing amino acids capable of buffering against pH change, or fat/lean tissue allowing for STEC to be removed from close proximity of free or micelle-encapsulated eugenol.

Others have also reported numbers of STEC on ground beef to be reduced only to a low extent following application of plant compounds with antimicrobial activity. The application of allyl isothiocyanate (AIT) via impregnated disc to ground beef reduced *E. coli* O157:H7 0.6 to 0.7 log_10_ CFU/g following five days’ storage at 4 °C under vacuum packaging [33]. Reductions in *E. coli* O157:H7 numbers on minced beef following application of plant-derived antimicrobial essential oil components and holding for 3 h at 5 °C approximated 0.1 to 0.5 log_10_-cycles [34]. Nonetheless, while some researchers have reported that application of plant-derived antimicrobials to ground beef produced no significant pathogen reduction, the application of these interventions enhanced lethality achieved during subsequent processing for inactivation of STEC or *Salmonella* [15,35].

## 4. Conclusions

The application of eugenol (free, micelle-encapsulated) to members of the O157 and non-O157 STEC in vitro was able to produce growth inhibition at MICs similar to those reported elsewhere. Nonetheless, the application of free or micelle-loaded eugenol to STEC-inoculated beef trimmings, as well as the application of 2% lactic acid (55 ± 1 °C) did not reduce STEC on trimming surfaces (Figure A1). Following grinding and five days of refrigerated storage, statistically significant, albeit biologically non-significant (≥1.0 log_10_-cycle) reductions in STEC were observed as a function of storage period (*p <* 0.05) (Figure 1). Eugenol was hypothesized capable of enhanced antimicrobial activity in the current study by its delivery to beef surfaces in an encapsulated form versus the unencapsulated plant-derived antimicrobial, reported elsewhere to occur in other high-protein animal-derived foods [17]. While this study is the first to the authors’ knowledge reporting the application of eugenol-loaded micelles for non-O157 STEC reduction on beef trimmings, analysis of experimental data leads authors to reject the original hypothesis that eugenol micelles would reduce STEC on beef trimmings versus controls when applied via spraying versus other methods. Beef safety researchers should continue investigating and optimizing post-harvest technologies for effective beef safety protection.

## Figures and Tables

**Table 1:** Shiga toxin-producing *Escherichia coli* (STEC) isolates, sources, and minimum inhibitory concentrations (MICs) of free and micelle-loaded eugenol.

STEC Serotype	Isolate ^1^	Source	Free Eugenol MIC (% *w/v*)	Micelle-Eugenol MIC (% *w/v*) ^2^
O157:H7	USDA-FSIS-380-94	Salami	0.5	0.125
O26:H11	H30	Clinical	0.5	0.125
O121:H19	CDC 97-3068	Human Stool	0.5	0.125

^1^ Isolates were provided by J.B. Luchansky (U.S. Department of Agriculture-Agricultural Research Service, Wyndmoor, PA, USA). ^2^ Micelles were comprised of sodium dodecyl sulfate (SDS; 1.0% *w/v*) by mixture of eugenol into aqueous dispersion of SDS, followed by stirring at ambient temperature. 1.0% SDS was the maximum non-inhibitory concentration (MNIC) of the surfactant against STEC isolates. USDA-FSIS: U.S. Department of Agriculture-Food Safety and Inspection Service; CDC: Centers for Disease Control and Prevention.

**Table 2:** Survival of Shiga toxin-producing *Escherichia coli* (STEC) on fresh beef trimmings following application of antimicrobial treatments.

Treatment ^1^	STEC Survivors (log_10_ CFU/g) ^2^	*p* > F	Pooled Standard Error
Sterile Water (25 °C)	6.5 ± 0.40	0.902	0.294
2× FreeEug	6.5 ± 0.36		
Micelles	6.4 ± 0.36		
2% LA	6.4 ± 0.25		

^1^ Treatments were: Control (inoculated, non-treated); Sterile Water (25 °C spray), 2× FreeEug (twice the minimum inhibitory concentration of eugenol applied by spray), Micelles (three times the minimum inhibitory concentration of micellarized eugenol in vitro, applied by spraying), and 2% LA (lactic acid solution, heated to 55 °C prior to spraying). ^2^ Values given depict mean STEC survivors from triplicate identical replications (*n* = 3) following inoculation, a 30 min attachment period, and spray-application of antimicrobial treatments ± one sample standard deviation from the mean. STEC mean inoculation on beef pre-treatment: 6.6 ± 0.40 log_10_ CFU/g.
